# Characterization and *In Vivo* Validation of a Three-Dimensional Multi-Cellular Culture Model to Study Heterotypic Interactions in Colorectal Cancer Cell Growth, Invasion and Metastasis

**DOI:** 10.3389/fbioe.2018.00097

**Published:** 2018-07-17

**Authors:** Sarah Cattin, Laurent Ramont, Curzio Rüegg

**Affiliations:** ^1^Department of Oncology, Faculty of Science and Medicine, Immunology and Microbiology, University of Fribourg, Fribourg, Switzerland; ^2^Laboratory of Medical and Molecular Biology, Centre National de la Recherche Scientifique, Reims, France; ^3^Swiss Integrative Center for Human Health, Fribourg, Switzerland

**Keywords:** colorectal cancer, three-dimensional model (3D), *in vitro*, invasion, tumor microenvironment, heterotypic interactions, animal use alternatives, 3R principles

## Abstract

Colorectal cancer (CRC) is the third cause of cancer-related mortality in industrialized countries. Local invasion and metastasis formation are events associated with poor prognosis for which today there are no effective therapeutic options. Invasion and metastasis are strongly modulated by cells of the tumor microenvironment (TME), in particular fibroblasts and endothelial cells. Unraveling interactions between tumor cells and cells of the TME may identify novel mechanisms and therapeutic targets to prevent or treat metastasis. We report here the development and *in vivo* validation of a 3D tumor spheroid model to study the interactions between CRC cells, fibroblasts and endothelial cells *in vitro*. Co-cultured fibroblasts promoted SW620 and HCT116 CRC spheroid invasion, and this was prevented by the SRC and FGFR kinase inhibitors Dasatinib and Erdafitinib, respectively. To validate these findings *in vivo*, we injected SW620 cells alone or together with fibroblasts orthotopically in the caecum of mice. Co-injection with fibroblasts promoted lung metastasis growth, which was fully reversed by treatment with Dasatinib or Erdafitinib. Co-culture of SW620 or HCT116 CRC spheroids with endothelial cells suppressed spheroid growth while it had no effect on cancer cell migration or invasion. Consistent with this *in vitro* effect, co-injected endothelial cells significantly inhibited primary tumor growth *in vivo*. From these experiments we conclude that effects on cancer cell invasion and growth induced by co-cultured TME cells and drug treatment in the 3D spheroid model *in vitro*, are predictive of *in vivo* effects. The 3D spheroid model may be considered as an attractive model to study the effect of heterotypic cellular interactions and drug activities on cancer cells, as animal testing alternative. This model may be adapted and further developed to include different types of cancer and host cells and to investigate additional functions and drugs.

## Introduction

After decades of experimental and molecular cancer research, drug development and testing, cancer remains a leading cause of death worldwide (Siegel et al., 2017b). Colorectal cancer (CRC) is the third cause of cancer-related mortality in industrialized countries (Brenner et al., 2014; Siegel et al., 2017a). Next generation DNA sequencing has allowed the identification of recurrent CRC mutations thereby opening the way to a better detection, classification and treatment of CRC (Dienstmann and Tabernero, 2016). Four consensus molecular subtypes (CMS) of clinical relevance have been recently reported based on RNA expression profiling (Guinney et al., 2015). At the cellular level, however, many questions remain unsolved, in particular those concerning mechanisms of invasion and metastasis, two of the main hallmarks of cancer (Hanahan and Weinberg, 2011; Valastyan and Weinberg, 2011; Sleeman et al., 2012). This is clinically highly relevant as cancer cell migration and local invasion are the first steps toward metastatic dissemination, which will eventually determine patient outcome (Brenner et al., 2014; McAllister and Weinberg, 2014).

Invasion and metastasis are not fully cell autonomous events. Multiple elements of the tumor microenvironment (TME) play critical roles not only in supporting local tumor growth but also in promoting invasion and metastasis in several cancer types, including CRC (Gout and Huot, 2008; Lorusso and Ruegg, 2008; Joyce and Pollard, 2009; Jeon et al., 2015; Malandrino et al., 2018). Growth factors production, metabolic changes, extracellular matrix (ECM) remodeling, activation of host cells such as fibroblasts, mesenchymal stem cells and endothelial cells as well as recruitment and polarization of immune and inflammatory cells, contribute to cancer growth, local invasion and distant metastasis formation (Lorusso and Ruegg, 2008; Quail and Joyce, 2013; Kalluri, 2016). As there are no robust, curative treatment for metastatic disease, the understanding of the mechanisms used by cells of the TME and their interaction with tumor cells to promote invasion and metastasis is essential for the rational development of new treatments impinging on local invasion and metastasis formation. The knowledge about the complex signaling circuits occurring between the tumor cells and the different cell populations of the TME is growing but still incomplete (Quail and Joyce, 2013; Bhome et al., 2015). Studying heterotypic cellular interaction *in vivo* is limited due to constrains in accessing the tissue, the simultaneous presence of multiple cell types, and the difficulty in selectively modulating specific cell types or intercellular interactions. In addition, *in vivo* monitoring requires invasive procedures and time-course experiments necessitate large amounts of animals (Taketo, 2006; Clarke, 2007; Golovko et al., 2015).

*In vitro* 2D co-culture models mimicking cancer-stromal cell interaction are widely used to identify new therapeutic targets and study new drugs. However, 2D tissue culture conditions do not mimic well *in vivo* heterotypic interactions, leaving a wide gap between *in vitro* and *in vivo* models (Bartlett et al., 2014). It is now generally accepted that 3D tissue culture is the preferred way of investigating cancer cells *in vitro* to bridge this gap. 3D tissue culture represents a more physiological setting to study morphology, cell cycle progression, cellular interactions, gene and protein expression, invasion, migration, and tumor metabolism. This is particular relevant to drug discovery and testing of anti-cancer agents as cells have different sensitivities in 3D vs. 2D conditions, including CRC cells (Stadler et al., 2015; Weiswald et al., 2015; Pereira et al., 2016; Penfornis et al., 2017; Ravi et al., 2017; Jin et al., 2018; Langhans, 2018). In addition, *in vitro* 3D co-culture models constitute invaluable tools to interrogate the role of individual cells of the TME and their interactions with cancer cells in tumor progression (Herrmann et al., 2014; Thoma et al., 2014; Horie et al., 2015; Ravi et al., 2015, 2017). We previously reported a 3D spheroid *in vitro* model of CRC to study multicellular interactions between tumor cells and fibroblasts and used it to decipher mechanisms by which fibroblasts promote CRC invasion (Knuchel et al., 2015). We showed that cell surface presentation of fibroblasts-derived FGF-2 to cancer cells, leads to integrin α_v_β_5_-dependent and SRC-mediated adhesion of cancer cells to fibroblasts, and contact-dependent tumor cell elongation, migration and invasion. Here we report the *in vivo* validation of results obtained with co-cultured fibroblasts and SRC and fibroblast growth factor receptor (FGFR) inhibitors in this 3D model *in vitro*. We also tested the effects of endothelial cells as additional cells to reconstitute the multicellular interactions in the TME.

## Results

### Dasatinib or erdafitinib treatment prevents fibroblast-promoted CRC cell migration and invasion *in vitro*

**Figure 1:**
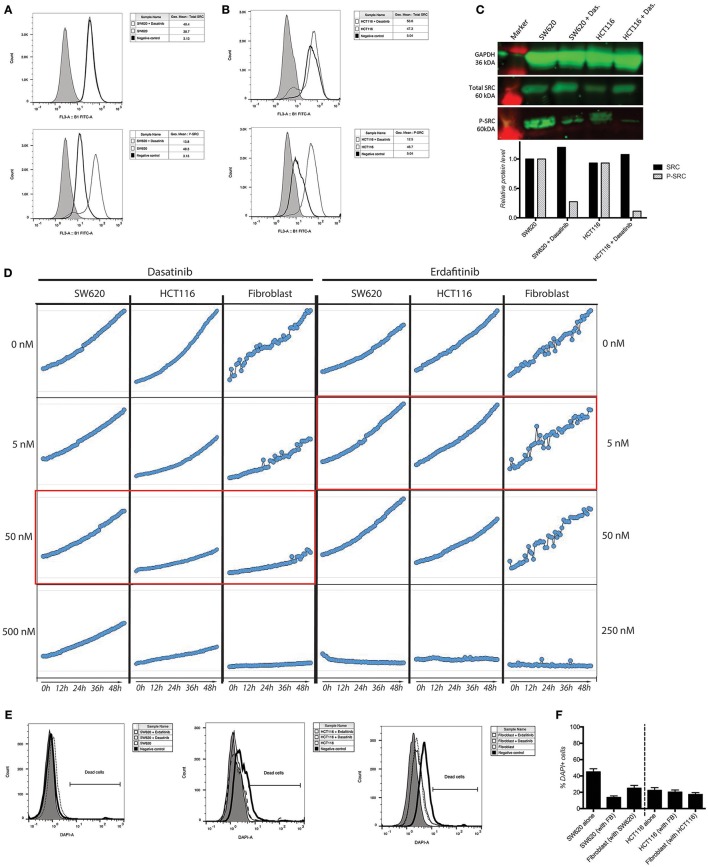
Activity and toxicity of Dasatinib and Erdafitinib. **(A,B)** Intracellular detection of total and phospho-SRC in SW620 **(A)** and HCT116 **(B)** show that Dasatinib inhibits SRC phosphorylation. **(C)** Western blot analysis confirms that Dasatinib suppresses SRC phosphorylation in cancer cells. **(D)** Growth curve of SW620 and HCT116 over 48 h in presence or absence of the described drugs at the described concentration. In red the *in vitro* used concentration for the two drugs. **(E)** Quantification of cell dead by flow cytometry after 7 days in 3D assay conditions. **(F)** Viability measurements of the different cell lines cultured in 2D conditions in the presence or absence of the corresponding inhibitor for 48 h using DAPI staining.

**Figure 2:**
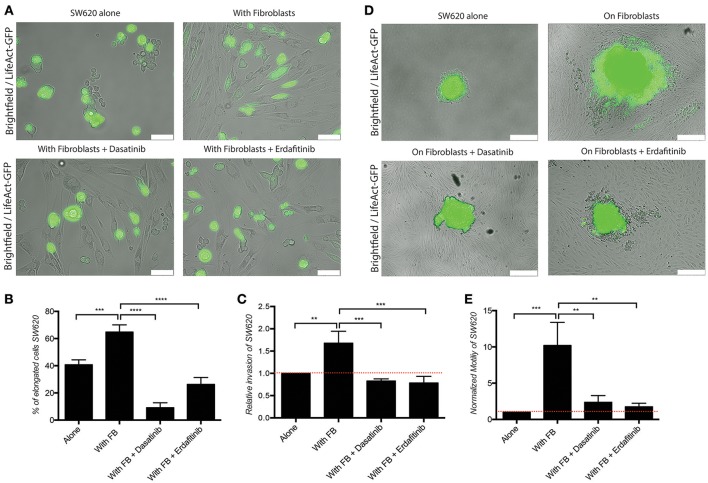
Dasatinib and Erdafitinib reduce fibroblasts-induced SW620 cancer cell elongation, migration and invasion *in vitro*. **(A)** Representative images of SW620-LifeAct-GFP cells cultured under 2D conditions with and without fibroblasts in the absence or presence of Dasatinib and Erdafitinib for 48 h. White bars represent 100 μm. **(B)** Quantification of elongation of SW620 cells of experiment in **(A)**, cultured as indicated, at day 4. **(C)** Quantification of SW620 cell spheroid 2D invasion under the indicated conditions after 4 days of culture. **(D)** Representative images of SW620-LifeAct-GFP cells of experiment in **(C)** cultured as indicated, at day 4. White bars represent 500 μm. **(E)** Quantification of motility of SW620 cancer cells cultured as indicated for 48 h. Both inhibitors block fibroblasts-induced SW620 elongation, migration and invasion. All quantification data represent mean values ± SD. **p ≤ 0.01, ***p ≤ 0.001, and ****p ≤ 0.0001. Red line represent control value at 1.

**Figure 3:**
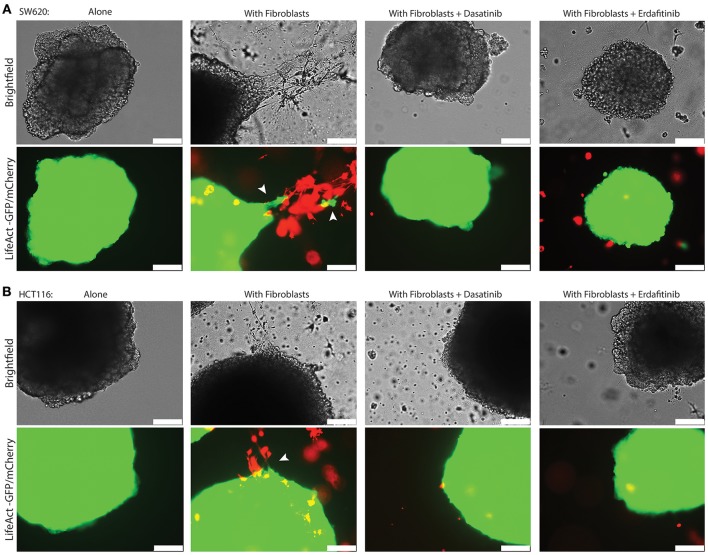
Dasatinib and Erdafitinib inhibitors reduce fibroblasts-induced SW620 cancer cell invasion *in vitro* under 3D condition. Representative images of **(A)** SW620-LifeAct-GFP and **(B)** HCT116-LifeAct-GFP 3D spheroid invasion with and without LifeAct-mCherry labeled fibroblasts in the absence or presence of Dasatinib and Erdafitinib inhibitors after 7 days. Both inhibitors are blocking fibroblasts-induced SW620 3D invasion. Arrows are indication the invasion area. White bars represent 250 μm.

We previously developed a 3D CRC cell-fibroblasts co-culture model and used it to demonstrate that fibroblasts promote contact-dependent cancer cell motility and invasion. Treatment with the SRC inhibitor CGP77675 and the FGFR inhibitor PD161570 prevented these *in vitro* effects (Knuchel et al., 2015). These results raised the question whether fibroblasts would also promote CRC invasion/metastasis *in vivo* in a SCR and FGFR-dependent manner. To test this hypothesis, we used two drugs in clinical practice or clinical development: Dasatinib, a BCR/ABL and SRC family tyrosine kinases inhibitor used to treat chronic myelogenous leukemia (CML) and acute lymphoblastic leukemia (ALL) (Lindauer and Hochhaus, 2014), and Erdafitinib, a potent pan-FGFR inhibitor (Perera et al., 2017) in clinical testing in advanced solid tumors, including breast, prostate, colon, bladder, esophageal and non-small-cell lung cancers (www.clinicaltrials.gov). Dasatinib reduced SRC phosphorylation (Figures 1A–C) in cancer cells and or Erdafitinib inhibited FGF-2 production in fibroblasts (Supplementary Figure S1). In drug titration experiments we identified non-toxic Dasatinib or Erdafitinib concentrations to use in the *in vitro* experiments (50 nM and nM, respectively, Figures 1D–F). Dasatinib or Erdafitinib treatment of SW620 and HCT116 CRC cells co-cultured with fibroblasts reduced fibroblast-induced cancer cell elongation, motility and invasion under 2D (Figure 2 and Supplementary Figure S2) and 3D conditions *in vitro* (Figure 3).

### Dasatinib or erdafitinib treatment prevents fibroblast-promoted CRC cell migration and invasion *in vivo*

**Figure 4:**
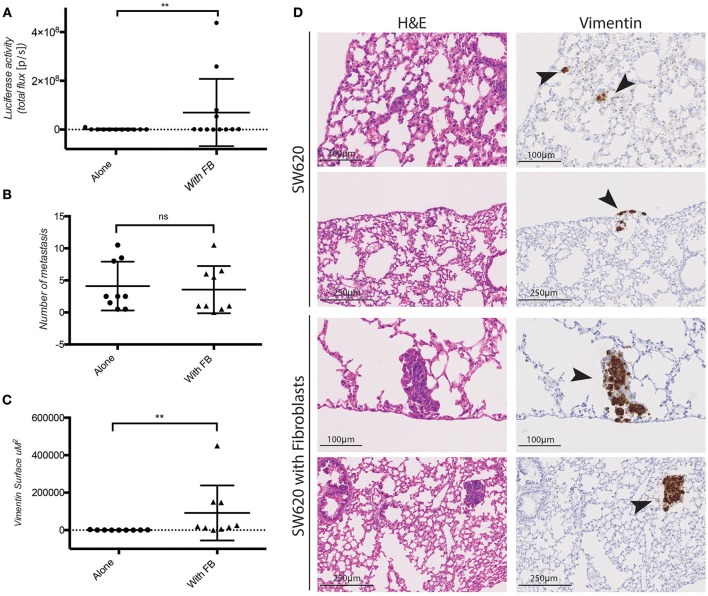
Fibroblasts promote SW620 colorectal cancer cell metastasis. **(A)** *Ex-vivo* Luciferase activity in the lung of mice injected with SW620-A299 ± fibroblasts. **(B)** Quantification of the number of metastatic nodules in the lung of mice orthotopically injected with SW620-A299 cells ± fibroblasts. **(C)** Quantification of the metastatic lung surface positive for vimentin by IHC of mice orthotopically injected with SW620-A299 cells ± fibroblasts. **(D)** Representatives images of consecutive sections of lungs of mice injected with SW620 ± fibroblasts stained by H&E (left) and for human vimentin by IHC (right). Arrows indicate metastases. Twelve mice per group were used. Quantification data represent mean values ± SD. Scale bars are given on the images. **p ≤ 0.01; Circles represent cells injected alone and triangles in presence of fibroblasts.

The pro-invasive effect of co-cultured fibroblasts *in vitro* raised the question whether fibroblasts co-injected with CRC cells would promote cancer metastasis *in vivo*. To this end SW620 cells expressing luciferase were orthotopically injected alone or together with fibroblasts in the caecum of immunocompromised (NSG) mice. Fibroblasts and tumor cells were mixed and injected at a 1:1 ratio which is a realistic approximation of the average Cancer Associated Fibroblasts (CAF): epithelial cell ratio observed in human CRC, considering variability observed in different CRC subtypes, stages and intertumoral heterogeneity (Henry et al., 2007; Isella et al., 2015; Nishishita et al., 2018). Fibroblasts co-injected with tumor cells did not impact primary tumor growth (Supplementary Figure S3). However, they induced a significant increase in the lung metastatic burden as detected by luciferase activity measured *ex vivo* (Figure 4A), and by immunohistochemistry (IHC) for human vimentin of the lungs (Figure 4D). Fibroblasts co-injection promoted the formation of larger metastatic nodules (Figures 4C,D), while it did not increase the number of the nodules themselves (Figure 4B).

**Figure 5:**
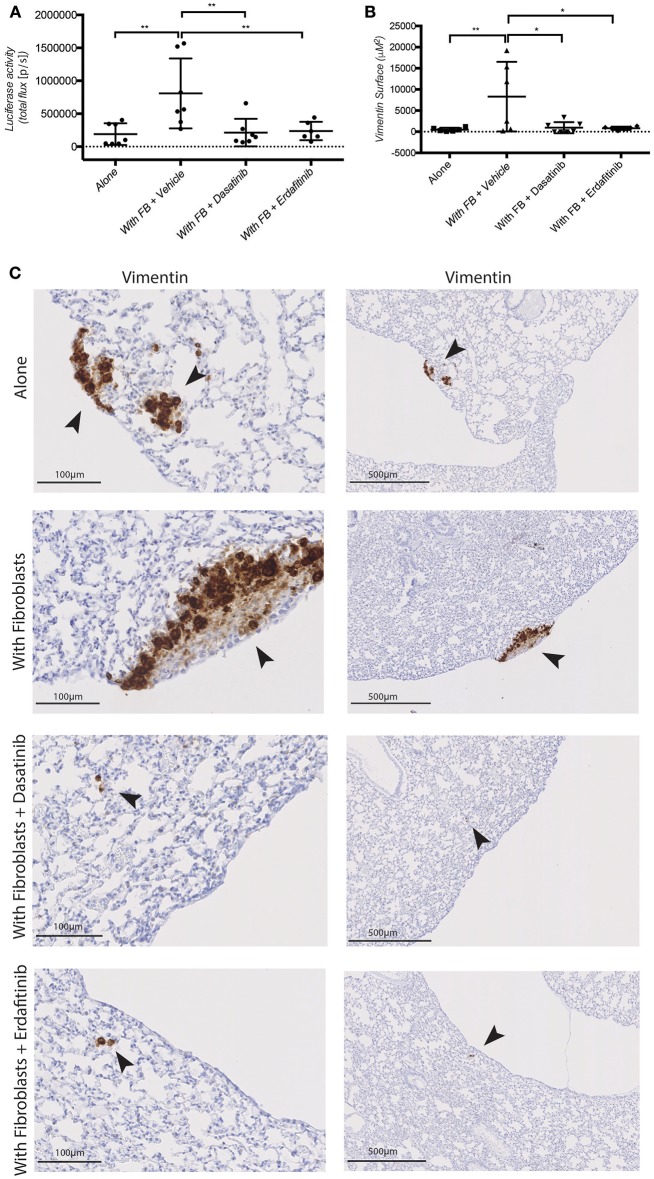
Dasatinib and Erdafitinib suppress fibroblasts-induced metastasis *in vivo*. **(A)** *Ex-vivo* Luciferase activity in the lung of mice orthotopically injected with SW620-A299 cells ± fibroblasts treated with Dasatinib or Erdafitinib or vehicle only as indicated. **(B)** Quantification of the metastatic lung surface positive by vimentin by IHC of mice of the same experiment. **(C)** Representatives images of consecutive sections of lungs of mice of the same experiment stained by IHC for human vimentin. Arrows are indication the metastases. Seven mice per group were used. Quantification data represent mean values ± SD. Scale bars are given on the images. *p ≤ 0.05, **p ≤ 0.01; Circles represent cells injected alone and triangles in presence of fibroblasts.

Next, we tested whether Dasatinib or Erdafitinib treatment would impinge on fibroblast-promoted metastasis formation *in vivo*, as it did for cancer cell migration and invasion *in vitro*. Treatments were started 2 weeks after primary tumor development was confirmed by *In Vivo* Bio Luminescence imaging (IBL; data not shown). Animal treated with Dasatinib or Erdafitinib showed a significant and homogeneous reduction of fibroblast-induced lung metastasis burden as detected by IBL imaging (Figure 5A). Immunohistochemical analysis of the lungs confirmed that Dasatinib and Erdafitinib treatment nearly completely reduced metastatic burden in the lungs as detected by IBL or by vimentin IHC of the lungs (Figures 5B,C). The number of metastatic lesions in the lungs, however, was not affected by the treatment (Supplementary Figures S4B–D). From week 4 on under treatment, animals were losing weight with both treatments (Supplementary Figure S4A), known side effect of kinase inhibitors due to intestinal toxicity (Galinsky and Buchanan, 2009), and therefore experiments were prematurely terminated at 6 weeks. As at this time point lung metastases in the treated animals are still microscopic, and therefore no significant increase in IBL signal was detected compared to untreated control animals in which lung metastases are already well established (Supplementary Figures S4B–D).

These data demonstrate that fibroblasts orthotopically co-injected with CRC cell promoted metastatic outgrowth (increased volume of metastatic lesions) in the lung, without however affecting the number of lesions. Conversely, treatment with the SRC and pan-FGFR inhibitors Dasatinib and Erdafitinib, respectively, prevented the fibroblasts-induced increase in lung metastasis. These *in vivo* results are consistent with *in vitro* results obtained with the 3D co-culture model, and further validate our previously *in vitro*-only study (Knuchel et al., 2015).

### Endothelial cells suppress CRC cell spheroid growth in the *in vitro* 3D co-culture culture model

**Figure 6:**
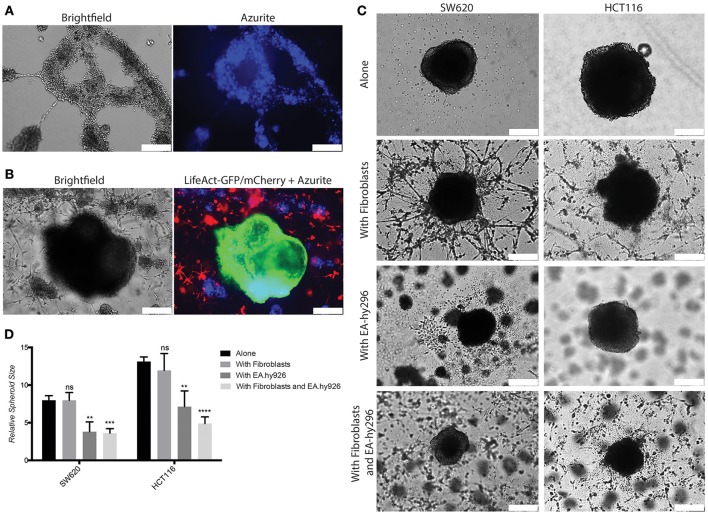
Co-culture with endothelial cells under 3D conditions inhibits SW620 and HCT116 spheroid growth *in vitro*. **(A)** Representative images of Ea.hy296 cells expressing Azurite cultured for 8 days under 3D conditions. **(B)** Representative images of HCT116-LifeAct-GFP cells cultured under 3D conditions with Ea.hy296-Azurite-cells and fibroblasts-LifeAct-mCherry cells. **(C)** Representatives brightfield images of SW620 and HCT116 colon cancer cells cultured for 7 days under 3D conditions in presence or absence of fibroblasts ± Ea.hy296 endothelial cells. **(D)** Quantification of relative size of SW620 and HT116 spheroids after 7 days of 3D culture. Quantification data represent mean values ± SD. White bars represent 500 μm. **p ≤ 0.01, ***p ≤ 0.001, and ****p ≤ 0.0001.

Based on these results we reasoned that this 3D model might be further developed to higher complexity by adding endothelial cells to mimic the vascular compartment of the TME. To this end we used an immortalized human umbilical vein endothelial cells (HUVEC)-derived cell line, Ea.hy296, previously reported to retain characteristics and functions of differentiated endothelial, such as angiogenesis, homeostasis/thrombosis, blood pressure and inflammation and are able to form a long lasting tube-like network *in vitro* (Edgell et al., 1983, 1990; Bauer et al., 1992; Rieber et al., 1993). We confirmed expression of the endothelial marker CD31 at similar level as in HUVEC (Supplementary Figure S5A). Ea.hy296 cells were genetically modified to express the life dye Azurite to assure visualization in the co-cultures (Figure 6A). As endothelial cells need a flat surface to form a visible tube-like network, the 3D model has been adapted accordingly by using a thicker second gel layer. 3D Co-culture experiments with CRC cells, fibroblasts and Ea.hy296 endothelial cells show that neither fibroblasts nor cancer cells directly interact with endothelial cells (Figures 6B,C). However, the presence of Ea.hy296 endothelial cells consistently reduced SW620 and HCT116 cell 3D spheroid growth (Figures 6C,D).

### Co-injected endothelial cells suppress cancer cell growth *in vivo*

**Figure 7:**
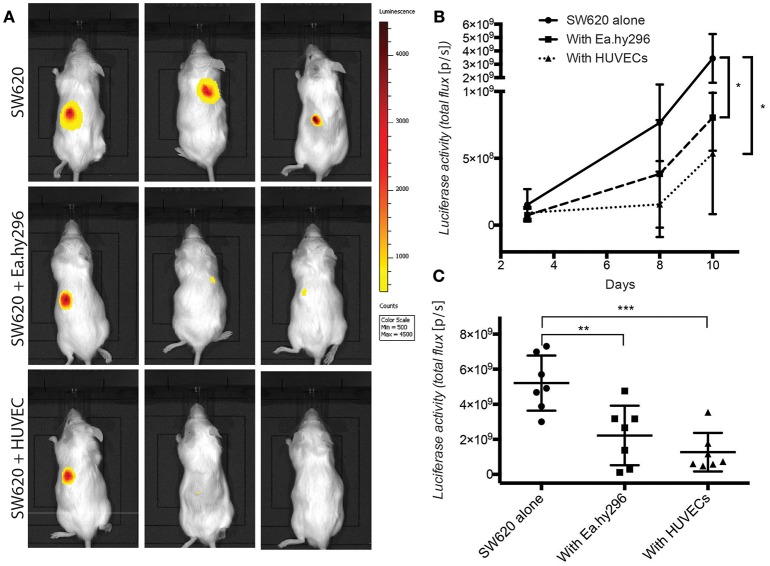
Co-injected endothelial cells reduce SW620 colon cancer growth *in vivo*. **(A)** Representative images of luciferase activity in mice subcutaneously injected with SW620-A299 in the presence or absence of EA.hy296 or HUVEC after 8 days. **(B)** Quantification of luciferase activity by IBL in mice subcutaneously injected at the indicated conditions over time. **(C)** Quantification of *ex-vivo* luciferase activity in tumors recovered 10 days after injection at the indicated conditions. Seven mice per group were used. Quantification data represent mean values ± SD. *p ≤ 0.1, **p ≤ 0.01 and ***p ≤ 0.001.

To validate these *in vitro* 3D results, Luciferase expressing SW620 and HCT116 CRC cells were injected subcutaneously in the back of NSG mice, alone and together with Ea.hy296 or HUVEC. Tumor growth was monitored over time by IBL and animals were sacrificed after 10 days. Co-injection of cancer cells with either Ea.hy296 or HUVEC significantly reduced the growth of both SW620 and HCT116 CRC cells (Figure 7 and Supplementary Figures S5B–D).

Taken together these results demonstrate that, in contrast to fibroblasts, which promoted spheroids invasion *in vitro* and metastasis formation *in vivo*, co-cultured and co-injected endothelial cells suppressed spheroids growth *in vitro* and tumor growth *in vivo*, respectively.

## Discussion

Local invasion and metastasis development in distant organs are the leading cause of cancer-related death (Siegel et al., 2017b). Mechanisms governing invasion and metastasis are only in part understood and the contribution of host cells at the primary and metastatic sites is being progressively unraveled (Quail and Joyce, 2013). Further understanding of these processes is essential in order to identify new mechanism and targets to develop novel therapeutic strategies to prevent tumor cell dissemination and outgrowth at distant sites. The multi-cellular nature of the TME and its multitude of soluble factors contributing to heterotypic communication, the changes in the extracellular matrix and the dynamic evolution of tumor-host complicate the *in vivo* study of the TME. In addition, animal models are expensive, time consuming, and subjected to higher variability (Heijstek et al., 2005; Clarke, 2007). Based on these considerations, we set up to develop a 3D heterotypic co-culture model allowing the study of the interactions between cancer cells and cells of the TME. Using this model, we previously reported that co-cultured fibroblasts promote CRC cells motility and invasion through a FGF-2/FGFR, α_v_β_5_-integrin, and SRC -dependent mechanism (Knuchel et al., 2015). The *in vivo* relevance of this pathway and its therapeutic implication were not assessed.

To prove that our 3D *in vitro* model is relevant to *in vivo* conditions, we performed *in vivo* experiments in mice in which we orthotopically injected SW620 and HCT116 CRC cells with or without fibroblasts. As a clinically relevant readout we monitored metastasis formation. Indeed, we observed that co-injected fibroblasts promoted the formation of larger metastatic nodules in the lungs, without, however, increasing the number of metastatic foci. Importantly, the larger metastatic burden induced by co-injected fibroblasts was prevented by inhibiting SRC and FGFR using Dasatinib and Erdafitinib, respectively. Consistent with the fibroblasts promoting effect, Dasatinib and Erdafitinib decrease the size of the lesions but not their numbers. Clinically, Dasatinib was tested in metastatic CRC patients, alone or in combination therapies, but showed no effect on disease progression (Sharma et al., 2012; Parseghian et al., 2017). These results are not comparable with our settings, as patients had already established metastases, while we tested the efficacy of SRC inhibition on the *de novo* formation of metastases. Our results indicate that SRC inhibition blocks metastasis formation when given early during disease progression. This setting would be comparable to an adjuvant treatment, for which to our knowledge, a clinical trial has not been reported yet. Through a gene-silencing approach, we have previously demonstrated that inhibition of SRC in tumor cells, but not in fibroblasts, is essential for the effect observed with pharmacological inhibition of SRC in the co-culture setting (Knuchel et al., 2015).

Taken together these *in vivo* results validate the *in vitro* results obtained with the 3D model at the cellular (i.e., fibroblasts promoted CRC cell invasion) and molecular (i.e., SRC and FGFR-depended fibroblast mechanism) levels. Although the endpoints of the two assays (i.e., motility and invasion *in vitro* vs. metastasis *in vivo*) are different, they are functionally linked as local invasion is considered as one of the first step of the metastatic cascade (Valastyan and Weinberg, 2011; Sleeman et al., 2012).

Next, we added endothelial cells to the 3D model to mimic the presence of a tumor-associated vasculature. Endothelial cells did not physically interact with fibroblasts or cancer cells and did not promote invasion but, unexpectedly, significantly decreased tumor spheroids growth. Consistent with these results *in vivo*, we indeed observed that tumor growth was decreased in animal co-injected with tumor cells and endothelial cells compared to tumor cells alone. Three dimensional models of tumor cells (spheroids)—endothelial cell interaction *in vitro*, have been largely used to study morphological and functions effect of tumor cells on endothelial cells including gene expression and sprouting angiogenesis, effect or angiogenic factors or anti-angiogenic drugs (Chopra et al., 1997; Khodarev et al., 2003; Van Moorst and Dass, 2011; Correa de Sampaio et al., 2012; Szot et al., 2013; Chiew et al., 2017; Wan et al., 2017). Co-culture with endothelial cells was shown to promote cancer cell (melanoma) invasion along tube likes structures (Yamamoto et al., 2014) or tumor cell growth when mixed inside spheroids (Upreti et al., 2011). However, in an on-top Matrigel model of ovarian cancer cells co-cultured with a tubular network of HUVEC, the presence of HUVEC clearly reduced tumor cell growth (Wan et al., 2017). No *in vivo* validation was performed in that work. The reason for this inhibitory effect remains elusive at this point and therefore these results should be carefully considered in their context. It is however tempting to speculate that quiescent, non-angiogenic endothelial cells may exert paracrine anti-proliferative/quiescence promoting effects to neighboring cells in contrast to pro-inflammatory and stimulatory effects of angiogenic endothelial cells (Potente et al., 2011). Furthermore, an important point to consider is that we used endothelial cells of macrovascular origin (HUVEC and a HUVEC-derived line) rather than microvascular endothelial cells from organs relevant to CRC, such as the colon itself (for the primary tumor) or the lung or liver for metastases. Considering the growing interest in understanding the role and activities of organotypic endothelial cells (Augustin and Koh, 2017; Potente and Makinen, 2017), this is an experimental aspect to include in follow-up experiments.

There are many different 3D assays described in the literature or available commercially (Kimlin et al., 2013b; Hoarau-Véchot et al., 2018), in the context of CRC (Nietzer et al., 2016; Pereira et al., 2016), vasculature modeling (Bersini et al., 2016; Haase and Kamm, 2017) and cancer invasion (Berens et al., 2015; De Jaeghere et al., 2018). The model that we proposed has been specifically developed to study the effect of cells of the TME, and in particular fibroblasts, on tumor cell motility and invasion. Globally, 3D *in vitro* models differ at two levels: firstly, on the preparation of the cancer spheroids. Hanging drop technique is the standard in creating spheroid either using manual pipetting or Bioprinting (Albritton and Miller, 2017). We used manual pipetting with a single spheroid per well. Secondly, models differ on the nature of the 3D matrix used. Both natural and synthetic matrices have been proposed and used for 3D culture conditions and invasion studies. For example, basement membrane extracts and hyaluronic acid are commonly used as biologically-derived matrixes; Polyethylene glycol (PEG), polyvinyl alcohol (PVA), polylactide-co-glycolide (PLG), and polycaprolactone (PLA) are common materials used to form synthetic scaffolds (Asghar et al., 2015; Chung et al., 2017). These matrices face similar limitations and potential criticisms, as they do not faithfully replicate the extracellular matrix of the TME. We opted for Matrigel, as there is ample literature on it and it is still the most widely used matrix in 3D assays, thereby allowing for direct for comparisons with other published reports (Benton et al., 2014).

In conclusion, we have developed a 3D *in vitro* heterotypic co-culture model to study CRC cell—stroma cell interactions. The model was validated for two CRC cell lines (i.e., SW620 and HCT116), two TME cell types (i.e., fibroblasts and endothelial cells) and two functions (i.e., growth and invasion/metastasis). This model may be adapted to different cancer types, such as breast or prostate, to include additional host cells, such as immune/inflammatory cells, microvascular endothelial cells of diverse organ origins, to test for additional functions, such as metabolism (Zecchin et al., 2015) or gene expression and under different conditions (e.g., hypoxia or starvation) (Bersini et al., 2016). This will help accelerating project progression and reducing the number of *in vivo* experiments and animal used (Kimlin et al., 2013a; Thoma et al., 2014).

## Materials and methods

### Cell culture

The human colorectal carcinoma cell lines SW620, HCT116, endothelial cells EA.hy296 and HEK-293T cells were purchased from ATCC (LGC Standards). Dermal fibroblasts were isolated from human neonatal foreskin as previously described (Knuchel et al., 2015). HUVEC were isolated from fresh umbilical cords dissociation and cultured as previously described (Yilmaz et al., 1998; Dormond et al., 2001). SW620 were cultured in RPMI GlutaMAXTM, HCT116, EA.hy296, fibroblasts and HEK-293T in DMEM GlutaMAXTM, all supplemented with 10% FCS, 100 U/ml Penicillin and 100 μg/ml Streptomycin. Cell detachment was performed using Trypsin-EDTA. Cell counting and viability determination was performed by trypan blue exclusion using Neubauer Counting Chamber. For co-culture experiments, fibroblasts and colon cancer cells were grown for 48 h at a 1:1 ratio in DMEM GlutaMAXTM. Growth curve to assess viability were monitored by Incucyte 10x. All cell culture reagents were purchased from Life Technologies. Protocols for collection and use of human samples were approved by the Ethic committees of Cantons Fribourg, Vaud, Berne and Ticino, Switzerland.

### Inhibitors of signaling

Dasatinib (Medkoo) was used *in vitro* at 50 nM in DMSO and *in vivo* at 30 mg/kg in 80 mM sodium citrate buffer pH 3.1 orally daily for 20 days. Erdafitinib (Medkoo) was used *in vitro* at 5 nM in DMSO and *in vivo* at 25 mg/kg in water with 1% Tween 80 orally daily for 20 days.

### Vectors and infections

LifeAct lentiviral vectors in GFP and mCherry were kindly provided by Dr. Olivier Pertz, Basel. A299 lentiviral vectors for Luciferase in GFP was kindly provided by Dr. Albert SantaMaria, Fribourg. pLV-Azurite plasmid was obtained from Addgene. Lentiviral particles were generated in HEK-293T cells by transducing the vector of interest with pMD2G (pSD11) and pMDLgpRRE (pSD16) plasmids using calcium phosphate transfection (Dull et al., 1998), followed, for LifeAct vectors, by an antibiotic-based selection of the infected cells and, for Azurite and A299 vectors, by a cell sorting using BD FACS ARIA Fusion based on GFP or blue fluorescent expression.

### 2D and 3D culture assay

Cancer cells spheroids were prepared as previously described (Kelm et al., 2003), using the hanging drop technic in a Terazaki plate with 500 cells per well in 20 μl medium for 72 h. For 2D spheroids assay, cancer cell spheroids were placed on top of a confluent fibroblasts layer. 3D spheroid assays were performed according to the 3D-On-Top method (Lee et al., 2007). Seven mg/ml Matrigel growth factor reduced (Corning) was used as matrix (Benton et al., 2014). Briefly, a first matrix layer was seeded on the well bottom of a non-treated multi-well plate to polymerize. A single spheroid was deposed on the layer and incubated 30 min at 37°C to adhere. A second matrix layer diluted 1:5 in culture medium was deposed on top of the spheroid. For 3D assay with fibroblasts, 75,000 fibroblasts/ml were added to the gel. For 3D assay with endothelial cells, the second layer on top of the spheroid was replaced by a 7 mg/ml Matrigel growth factors reduced matrix, mixed or not with 75,000 fibroblasts/ml. After 45 min polymerization 150,000 Ea.hy296 were seeded on top in DMEM. Time course of the *in vitro* experiments is given in Supplementary Figure S6A.

### *In vivo* experiments

Male NOD/SCID common gamma chain (IL-2Rγ^−/−^) ko (NSG) animals between 7 and 8 weeks old were used for *in vivo* experimentation and were purchased from the University of Lausanne. Twelve or 7 mice per group were used, depending on the expected difference of 40 or 50% or more for a SD of 30%, alpha = 0.05, and beta = 0.8. Orthotopic injection in the caecum was performed as previously described (Céspedes et al., 2007; Ragusa et al., 2014). The number of tumor cells was kept constrain in all conditions (i.e., ± fibroblasts) in accordance with widely accepted protocols (Picard et al., 1986; Erez et al., 2010; Li et al., 2012; Shintani et al., 2013; Cohen et al., 2017; Nakaoka et al., 2017; Wang et al., 2017). Briefly one million SW620-A299 cells, mixed or not with one million fibroblasts, resuspended in 50 μl DMEM solution containing 50% Matrigel were injected using a 30G needle in the submucosa of the caecum under a binocular loupe. The 1:1 fibroblasts: tumor cell ration was chosen as a realistic approximation of the average CAF: epithelial cell ratio reported for human CRC (Henry et al., 2007; Isella et al., 2015; Nishishita et al., 2018). Tumor growth was monitored by *in vivo* bioluminescence after i.p. injection of 1.5mg/g luciferin (Biosynth), using IVIS Lumina II (Xenogen). Sub-cutaneous tumor transplantation model were performed by injecting subcutaneously in the back a cell suspension of one million SW620-A299 cells or HCT116-A299, mixed or not with one million Ea.hy296 in 50 μl DMEM solution containing 50% Matrigel. Animal procedures were performed in accordance with the Swiss and French legislations on animal experimentation and approved by the Cantonal Veterinary Service of the Canton Fribourg (2016_06_FR). A schematic of the experiment is represented in the Supplementary Figure s6B.

### Immunohistochemistry

Slide staining's were performed on Leica BOND RX system following manufacturer's instructions. Paraffin embedded organ cuts were pre-treated 15 min with 1% H_2_O_2_ in Methanol for vimentin staining. Primary antibody (Dako) was incubated for 40 min at 1:100.

### Flow cytometry

For viability assay, cultures were harvested on ice and resuspended in PBS containing 3% FCS and 5 mM EDTA. DAPI staining (Miltenyi) was used at 1/1,000 dilutions 5 min before analysis. For 3D recovery and viability testing, cells were dissociated from recovered spheroids using the BD Recovery Solution (BD Biosciences) following manufacturer instructions.

For endothelial cells characterization an anti-CD31-Alexafluor 647 (BD Biosciences) was used at 1/100 dilution.

For intracellular staining, cells were fixed and permeabilized using a BD Bioscience Fix/Perm assay following manufacturer instructions. Anti-SRC, phospho-SRC and FGF-2 unlabeled primary antibodies (Cell Signaling) were used 30 min at 1/100 dilution and secondary anti rabbit-Alexa Fluor 488 (Cell Signaling) was used at 1/300 for 30 min. A MACSQuant instrument was used to perform experiments and FlowJo 10.4 (Treestar Inc.) software was used to analyze all data.

### Western blotting

Cells were lysed using RIPA lysis buffer (Cell Signaling) supplemented with protease Cocktail inhibitor (Sigma), 2 mM PMSF, 2 mM BGE (Sigma) and 0.2 mM Orthovanadate (Sigma) on ice and re-suspended in SDS buffer containing 10% Glycerol, 5% β-mercatoethanol, 60 mM Tris-Cl, trace of bromophenol blue and 2% SDS (Sigma-Aldrich). SDS-PAGE, blotting, and detection were performed as previously described using a Lumina Odyssey instrument.

### Imaging and data analysis

Imaging of 2D and 3D spheroids assay were performed with 5x and 40x objectives with Leica AF6000 inverted fluorescence microscope. For 3D assay, spheroid growth was quantified using Photoshop CS6 (Adobe) by calculating the spheroid size area on the 7 days image, normalized on initial spheroid size. For 2D assay, invasion was quantified using Photoshop CS6 (Adobe) by calculating the invaded area on image taken at day 4 and normalized on the initial spheroid size. For co-culture experiments images were taken every hour at different magnifications with an inverted fluorescence microscope (Leica AF6000). Cancer cell elongation was quantified manually by scoring individual cells using Image J “Cell Counter” plugin on taken images at 48 h co-culture. Elongated cells were defined as single cells with a minimal length-width rapport of 2:1, and at least one sharp-ending extremity. Cancer cell motility was quantified based on single cell track follow for 24 h on time-lapse movies using Image J “Manual Tracking” and “Chemotaxis Tool” plugins. Western Blot images were quantified for protein level using Image J “Gel Analyze Tool” plugin. For immunohistochemistry images, slides were scanned using a NanoZoomer 2.0 HT (Hamamatsu) and images were extracted and analyzed using NDP.view2 analysis software (Hamamatsu). Metastases were counted based on the presence of vimentin positive staining in the lung. Each isolated positive cell or cell cluster (touching) was considered as a metastasis.

### Statistical analysis

Each experiment was repeated independently a minimum of 3 times in triplicate conditions. Acquired data were analyzed using Prism Software (GraphPad). Statistical comparisons were performed by un-paired two-tailed Student's *t*-test or by two-way ANOVA with Bonferroni post-test. Results were considered to be significantly from *p* < 0.05.

## Author contributions

SC planned and designed the project; designed and performed experiments; analyzed data; wrote the manuscript. LR designed and performed part of the experiments on endothelial cells; analyzed data. CR planned and designed the project; evaluated experiments; wrote the manuscript.

### Conflict of interest statement

The authors declare that the research was conducted in the absence of any commercial or financial relationships that could be construed as a potential conflict of interest.
