# Supplementary material for: Characterization and In Vivo Validation of a Three-Dimensional Multi-Cellular Culture Model to Study Heterotypic Interactions in Colorectal Cancer Cell Growth, Invasion and Metastasis
Source: Front Bioeng Biotechnol. 2018 Jul 17;6:97. doi: 10.3389/fbioe.2018.00097 (PMC6056662; doi:10.3389/fbioe.2018.00097)
Supplement: Supplementary file 1 [file Image_1.pdf]

Supplementary Material

Characterization and in vivo validation of a three-dimensional multi-cellular culture model to study heterotypic interactions in colorectal cancer cell growth, invasion and metastasis

Sarah Cattin[1], Laurent Ramont[2] and Curzio Rüegg[1*]

*** Correspondence:** Curzio Rüegg: curzio.ruegg@unifr.ch

Supplementary Figures

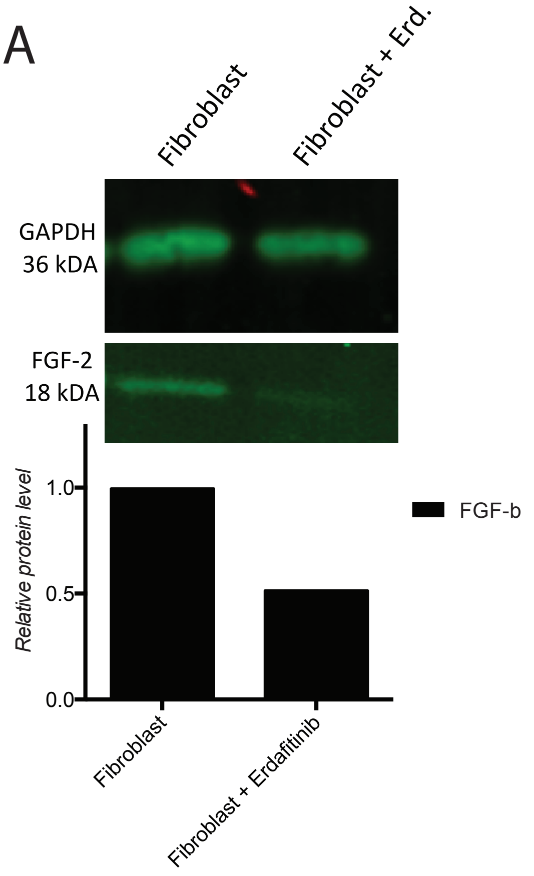

Supplementary Figure S1. Erdafitinib activity on fibroblasts. (A) Western blot quantification of FGF-2 protein level in fibroblasts cultured in the absence or presence of Erdafitinib for 24 hours.

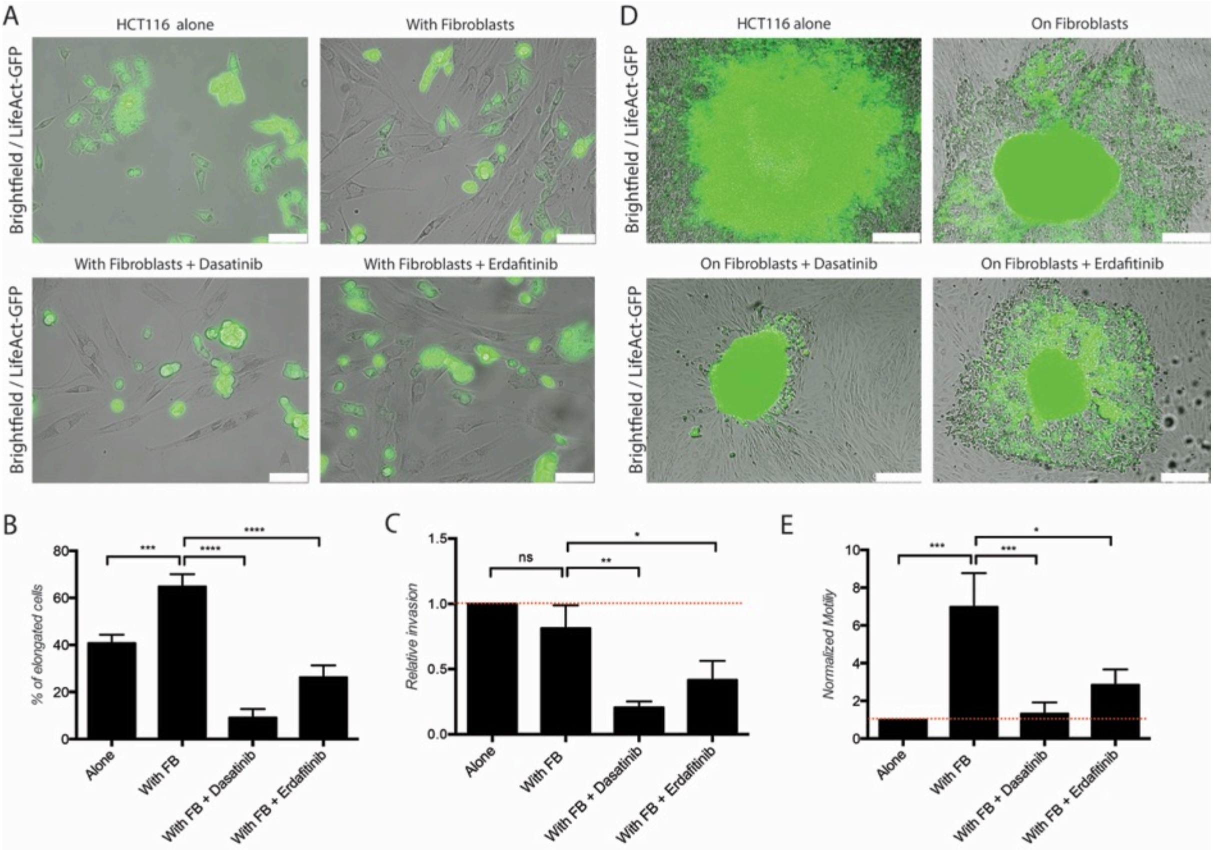

Supplementary Figure S2. Dasatinib and Erdafitinib reduce fibroblasts-induced HCT116 cancer cell elongation, migration and invasion *in vitro*. (A) Representative images of HCT116-LifeAct-GFP cells under 2D conditions with and without fibroblasts in the absence or presence of Dasatinib and Erdafitinib for 48 hours. White bars represent 100 µm. (B) Quantification of elongation of HCT116 cells of experiment in panel A, cultured as indicated, at day 4. (C) Quantification of HCT116 cell spheroid 2D invasion under the indicated conditions after 4 days. (D) Representative images of HCT116-LifeAct-GFP cells of experiment in panel C cultured as indicated, at day 4. White bars represent 500 µm. (E) Quantification of motility of SW620 cancer cells cultured as indicated for 48 hours. Both inhibitors block fibroblasts-induced SW620 elongation, migration and invasion. All quantification data represent mean values +/- SD.

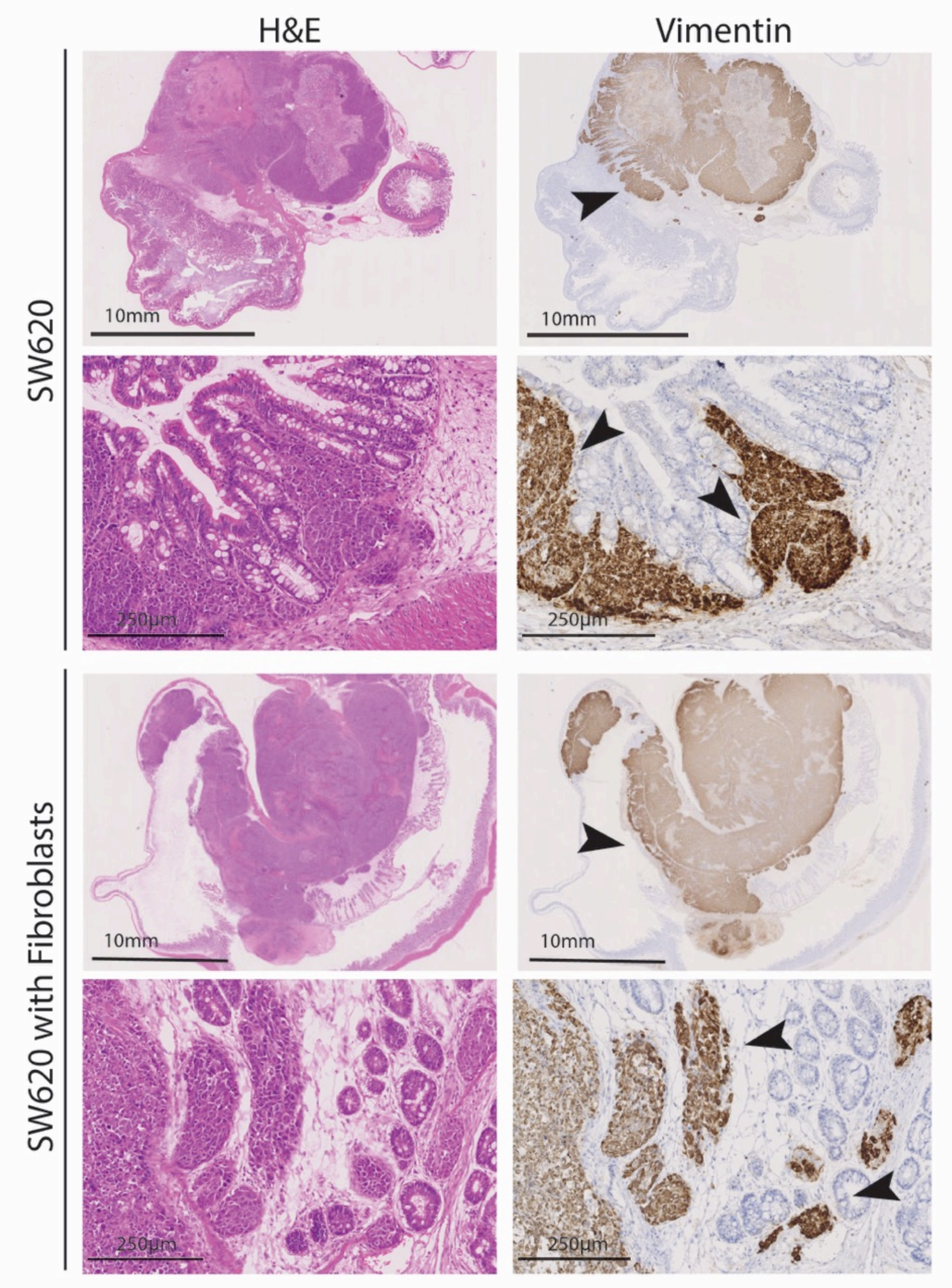

Supplementary Figure S3. Histological analysis of primary tumors. Representative images of consecutive sections of primary colorectal tumor of mice orthotopically injected with SW620 +/- fibroblasts and stained by H&E (left) and for human vimentin by IHC (right). Scale bars are given on the images.

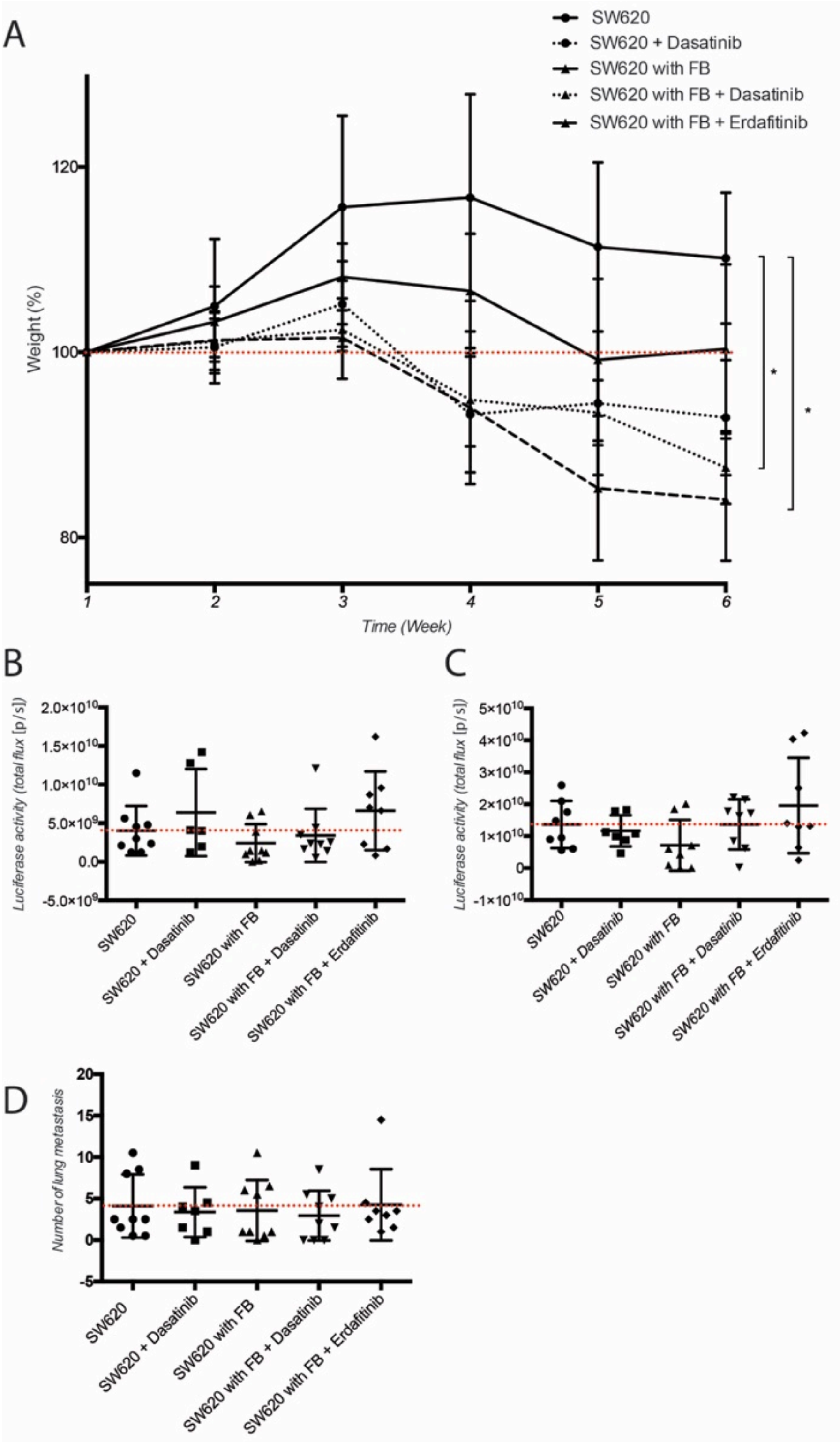

Supplementary Figure S4. ***In vivo*** **effects of Dasatinib and Erdafitinib inhibitors.** (A) Animal weight follow-up over experimental time. From week 4 treated amice start losing weight leading to premature termination of the experiment (B) Quantification of total body luciferase activity at the end of the treatment in mice injected and treated as indicated. (C) Quantification of *ex-vivo* colon tumor luciferase activity at the end of the treatment in mice injected and treated as indicated. (D) Quantification of number of metastasis in the lung at the end of the treatment in mice injected and treated as indicated. All data are represented as mean +/- SD.

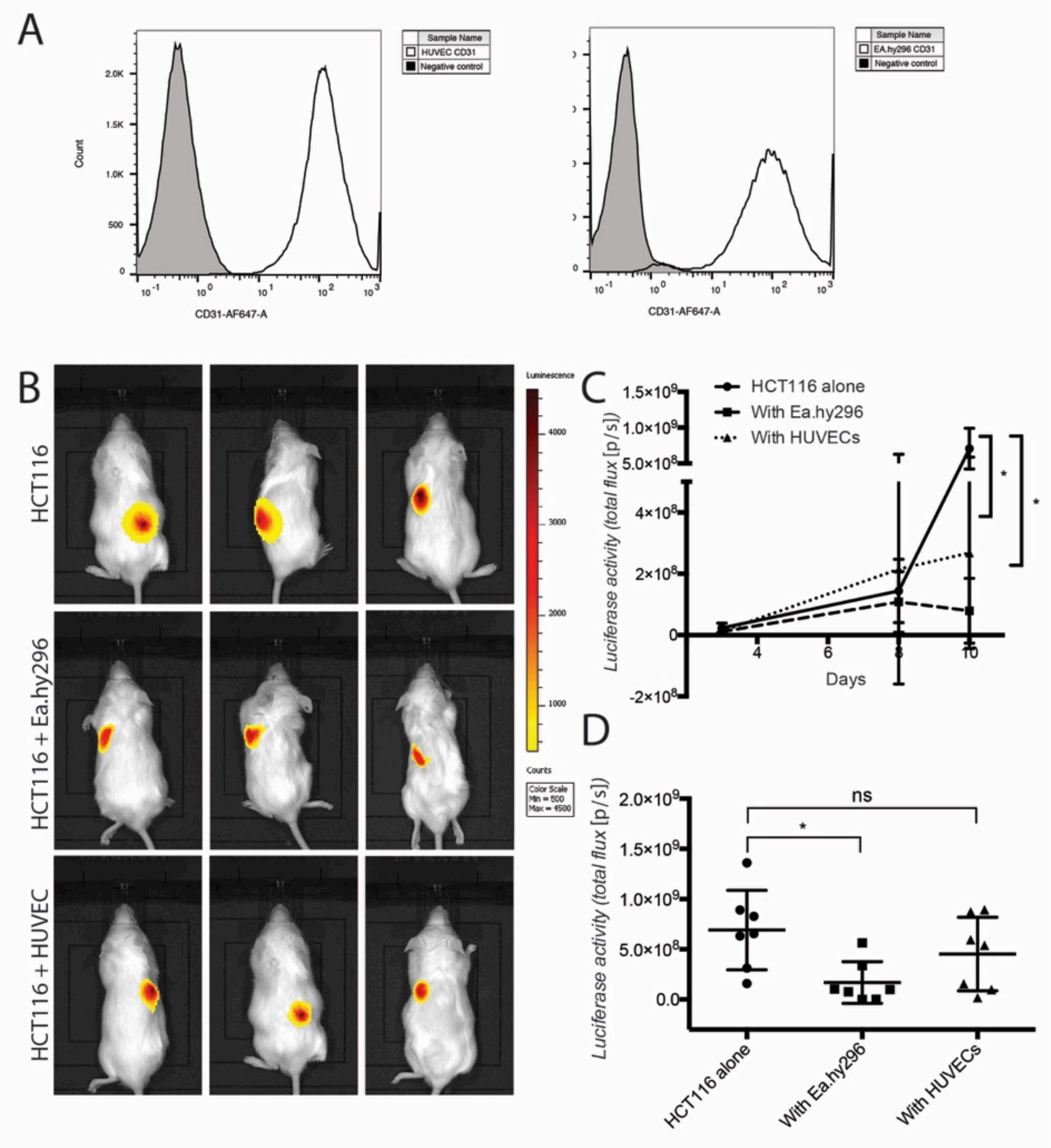

Supplementary Figure S5. Co-injected endothelial cells reduce HCT116 colon cancer growth *in vivo*. (A) Characterization of HUVEC and Ea.hy296 by CD31 expression. (B) Representative images of luciferase activity in mice subcutaneously injected with HCT116-A299 in the presence or absence of EA.hy296 or HUVEC after 8 days. (C) Quantification of luciferase activity in mice subcutaneously injected at the indicated conditions over time. (D) Quantification of *ex-vivo* luciferase activity in tumors recovered 10 days after injection at the indicated conditions. Quantification data represent mean values +/- SD.

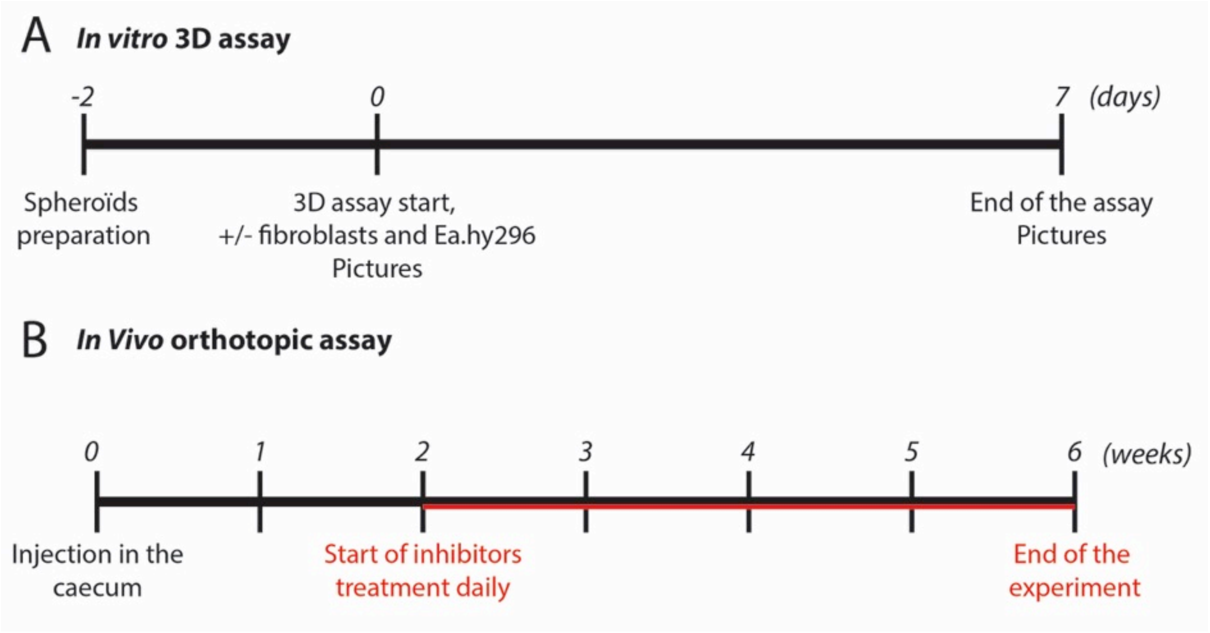

Supplementary Figure S6. Schematic of the experiments time-course. (A) Time-line of the 3D culture experiments. (B) Time-line of the orthotopic in vivo experiments.
